# Spatiotemporal characteristics of soil erosion in a typical watershed consisting of different landscape: A case study of the Qin River Basin

**DOI:** 10.1371/journal.pone.0275470

**Published:** 2022-10-03

**Authors:** Yanyan Li, Tianqi Rong, Mingzhou Qin, Pengyan Zhang, Dan Yang, Zhenyue Liu, Ying Zhang, Hui Zhu, Meiling Song

**Affiliations:** 1 College of Geography and Environmental Science, Henan University, Kaifeng, China; 2 Henan Overseas Expertise Introduction Center for Discipline Innovation (Ecological Protection and Rural Revitalization Along the Yellow River), Henan University, Kaifeng, China; 3 Key Laboratory of Geospatial Technology for the Middle and Lower Yellow River Regions (Henan University), Ministry of Education, Kaifeng, China; 4 Regional Planning and Development Center, Henan University, Kaifeng, China; Universidade Federal de Uberlandia, BRAZIL

## Abstract

Soil erosion has a severe impact on habitat and productivity. It is considered to be a major environmental threat prevalent in ecosystems. However, few researchers have studied the spatial distribution of soil erosion intensity among different geographic environmental factors. The Qin River Basin is a geographical unit consisting of mountains, hills, and plains with significant regional characteristics, and it has a basin area of 14,810.91 km^2^. This study uses the Geographical Information Systems, Revised Universal Soil Loss Equation model to analyze the spatiotemporal changes in the soil-erosion intensity in the Qin River Basin from 1990 to 2018. Different environmental factors of land use, slope and altitude on erosion intensities of 19 secondary land types were analyzed. It can better reflect the soil erosion under different environmental factors and different land use types. Results show that the soil erosion modulus of Qin River Basin were 10.25 t hm^−2^ a^−1^, and it belong to slight erosion from 1990 to 2018. Soil erosion intensity is greater in grassland and woodland than in cropland. The strongest soil erosion occurred in the sparse forestland, and the lowest was in beach land. Soil erosion was the highest for a slope of 15~25° and an altitude of 1200~1500 m. Rainfall and slope are important factors lead to soil erosion, indicating weak water and soil conservation implemented in these areas. Therefore, priority should be given to these geomorphic units to formulate and implement soil-erosion control and ecological restoration policies in the Qin River Basin. This study provides a good reference for preventing and controlling soil erosion in river basins.

## 1. Introduction

Soil erosion affects 56% of the land surface worldwide and directly threatens regional ecological security and sustainable economic and social development [1,2]. It leads to the destruction of land resources, restricts food production, increases disaster frequency, reduces soil fertility, pollutes water resources, silts up reservoirs and lakes, endangers cities, and causes a series of ecological and environmental problems [3–5]. Soil characteristics, climate, topography, and human activities have an important effect on soil erosion [6–8]. Borrelli et al. pointed out that global soil erosion increased by 20% from 2001 to 2012 and caused degradation to approximately 38% of agricultural land [9]. The soil replacement cost in Europe is estimated at 20 billion US dollars per annum [10]. The average rate of soil erosion in South America is 46%. In 2018, the area of soil erosion was 294.9 × 10^4^ km in China [11]. Soil erosion has seriously threatened the country’s ecological security and sustainable development. Climatic factors have an important influence on the impact of soil erosion. In particular, the effects caused by high winds and heavy rainfall, etc. are more direct. The elements of rainfall include rainfall intensity, rainfall amount, rainfall type, rainfall ephemeris, rain drop size, etc. Wind is the direct driving force that causes wind and sand flow and leads to soil wind erosion. The strength of wind erosion depends on the magnitude of wind speed and the structural condition of the ground composition material. Influenced by the ground friction resistance, the closer to the ground, the smaller the wind speed, the stronger the turbulence and eddy action; the higher the distance from the ground, the higher the wind speed, the airflow is also more stable. Wind and rain together drive the occurrence of soil erosion. Hence, Special soil protection strategies urgent need to be adopted. By analyzing the spatial characteristics of soil erosion patterns, we can directly display the regional distribution characteristics of areas with high erosion intensity. This information can help the choice of important areas to control soil erosion and optimize control measures. Research on soil erosion has gathered momentum owing to the increasing attention to environmental problems [12–14]. Estimation of soil erosion at the catchment scale helps in understanding its mechanism and sets the foundation for formulating soil and water conservation planning, restoration, and reconstruction measures [15,16].

Since the end of the 19th century, researchers have established many empirical statistical and physical models at various scales to understand the types, mechanisms, and spatiotemporal distribution of soil erosion to determine erosion-prone areas [17–19]. Examples of physical models include the Water Erosion Prediction Project (WEEP) [20], European Soil Erosion Model (EUROSEM) [21], and Limburg Soil Erosion Model (LISEM) [22]. Compared to physical models, empirical statistical models have a simple structure with strong applicability. They include the Universal Soil Loss Equation (USLE) [23], Revised Universal Soil Loss Equation (RUSLE) [24], and Chinese Soil Loss Equation (CSLE) [25]. Of these, RUSLE considers the factors that influence soil erosion using minimal data and calculation requirements, thereby allowing the quantitative extraction of spatial features. The RUSLE model focuses on the combined superposition of six factors, including rainfall erosivity factor (R factor), soil erodibility factor (K factor), slope length factor (L factor), steepness factor (S factor), cover management factor (C factor) and conservation practices factor (P factor), to obtain regional soil erosion [26]. The soil erosion process in a vast area and its spatial distribution characteristics at different time periods under various scenarios can be quickly evaluated and simulated through a combination of Geographical Information Systems (GIS) and soil-erosion assessment model [27]. Traditional research methods such as the runoff-plot method and rainfall-erosion simulation method cannot obtain soil erosion data of the region promptly and will hinder or ‘slow down’ water conservation measures by ecological management. Therefore, many scholars have conducted research on the status, influencing factors, sensitivity analysis, and soil erosion prediction at different scales using the RUSLE model [28–31]. Peng and Wang conducted the hillside surveillance field test in Southwest China, and proved that the larger the rainfall, the greater the amount of soil loss [32]. Wang et al. revealed that vegetation coverage is the main driving force for soil loss in the Yellow River source area of the Qinghai-Tibet Plateau [33].

The RUSLE model can accurately evaluate soil erosion and water loss statuses of a river basin. The diversity of terrain and climatic conditions in the Qin River Basin cause frequent drought and flood disasters. Furthermore, human activities, such as ecological resettlement, engineering development, and construction threaten the vegetation and habitats in this study area. The Qin River Basin is a large geographic unit that consists of mountains, hills, and plains. More than half of the basin consists of mountainous forest areas, one-third comprise rocky hills, and approximately one-tenth is wetlands. The region has a unique culture and landscape and has rich animal and plant resources. The basin has a complex and diverse ecosystem. The sources of the rivers and the rivers in the loess hilly area are typical with distinct seasonal characteristics. The Qin River is also an important water source for the Shanxi and Henan provinces and plays a critical role to the lives and production of the area. Previous studies on soil erosion were conducted under similar environmental conditions, which provided a limited scientific basis for various land managers and policy makers. Research on the spatiotemporal characteristics of soil erosion has important theoretical and practical significance for formulating targeted soil and water conservation measures and improving the ecological environment.

Previous studies have mainly focused on the assessment of soil erosion, regional spatial and temporal variability and soil erosion in a single ecosystem type, and integrated comparisons between geographic elements are still lacking. In addition, the effects of single factors on soil erosion are mainly analyzed, while the spatial distribution of different soil erosion intensities is more important guidance for land managers and decision makers compared to the estimation of soil erosion. Soil erosion in a watershed is a complex process influenced by natural factors and human activities, such as rainfall, topographic conditions, soil characteristics, vegetation characteristics and land use management. The vegetation, slope, and land use types vary depending on the development stage of the watershed geomorphology. How to quantitatively describe the relationship between its elevation, land use type, slope and vegetation cover and to explore the relationship between soil erosion intensity and vegetation cover, land use type and slope through the assessment of watershed geomorphic features is of great significance to solve the important scientific problem of industrial restructuring and soil erosion management in the current watershed.

There are few researches on soil erosion in the Qin River Basin. It is important to assess soil erosion in the Qin River Basin because of the increase in population and human activities such as overgrazing and reclamation. This study combines the RUSLE model with GIS technology and uses data on land use, Normalized Difference Vegetation Index (NDVI), daily precipitation and related soil texture, and Digital Elevation Model (DEM) to study the variations in soil erosion in the Qin River Basin from 1990 to 2018. This study is expected to offer a scientific basis for the soil and water loss conservation with respect to the appropriate allocation of land use and the formulation of relevant policies in the Qin River Basin. This study can be used as a reference for other river basins of the world with similar geomorphic features consisting of mountains, hills, and plains.

The purpose of this study is to explore the soil erosion characteristics of different geographic elements of soil erosion in the Qin River Basin. The specific objectives are: (1) to reveal the soil erosion changes of different land use types according to the secondary land use classification criteria; (2) to explore the distribution pattern of soil erosion intensity in the Qin River Basin and visually demonstrate the regional distribution location characteristics of areas with high erosion intensity; (3) to quantitatively analyze the magnitude of erosion intensity of different geographic elements based on soil erosion intensity and determine the priority areas of soil erosion in the basin.

## 2 Materials and methods

### 2.1 Study area

**Fig 1:**
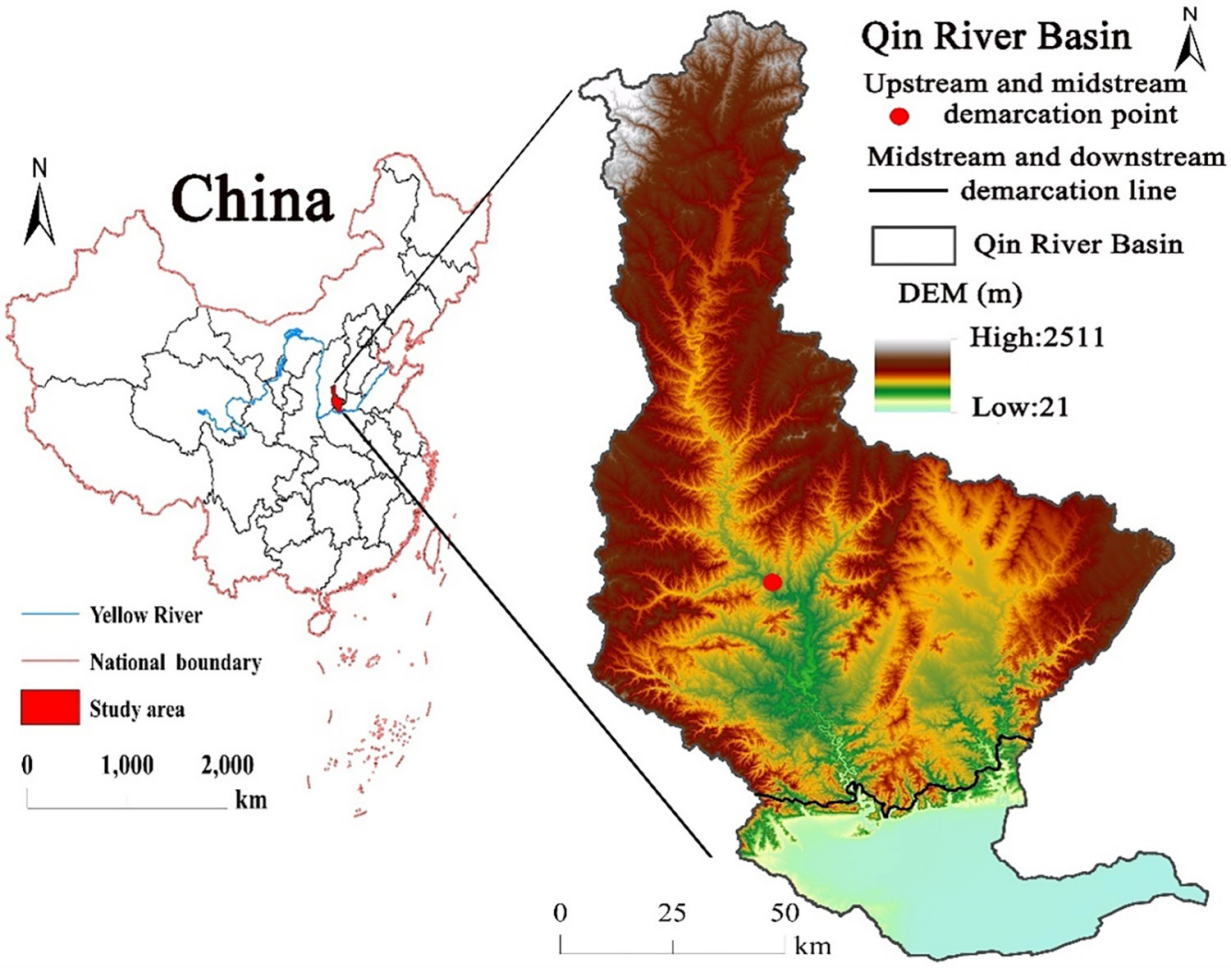
Location map of the Qin River watershed. DEM was obtained from National Tibetan Plateau Data Center (http://data.tpdc.ac.cn). Reprinted from http://data.tpdc.ac.cn under a CC BY 4.0 license, with permission from National Tibetan Plateau Data Center, original copyright [2019]. The national boundary was obtained from Natural Earth (http://www.naturalearthdata.com/). The scope of the Qin River Basin was determined based on DEM and eight-direction (D8) algorithm. Based on authors’ field investigation, we have modified and adjusted the vector boundary.

The Qin River Basin belongs to Shanxi and Henan provinces (111°55’E–113°30’E and 35°11’N–37°08’N). It is a first-level tributary of the Yellow River. The scope of the Qin River Basin was determined based on DEM and eight-direction (D8) algorithm. We used the hydrology tools in ArcGIS10.2 software based on the downloaded DEM data to obtain the basin extent of the Qin River basin after filling depression, flow direction, flow accumulation, basin, snap pour point and watershed tools, and the vector boundary of the Qin River basin after raster transect. The Chinese vector map was obtained from Natural Earth, and finally the location map of the study area was obtained by the cartography tool of ArcGIS 10.2 software. Its main stream has a length of 485 km. And its basin area is 14,810.91 km^2^ (Fig 1).

The Qin River Basin belongs to continental monsoon climate. Its annual average precipitation is 613.1 mm and average temperature is 5–11°C. The upper reaches (from the river source to Zhangfeng Reservoir) consist of rocky mountains with steep slopes and dense forests. Vegetation still retains the original natural landscape, and it is dominated by natural forests with tree species. The middle reaches extends from the Zhangfeng Reservoir to the boundary of Shanxi and Henan provinces, China. It is a hilly region of earth and rock, and the vegetation cover is lesser than that of the upper reaches. Vegetation types include mixed ever-green and deciduous forests and shrub grasslands. The lower reaches (Henan section, China) have a flat terrain and are dominated by agricultural production and irrigation development.

### 2.2 Data sources

The data included DEM, soil, climate, land use, and NDVI. Soil erosion factors were obtained using ArcGIS10.2, Anusplin 4.3, and ENVI 5.3. ArcGIS 10.2 software is a GIS software developed by the Environmental Systems Research Institute in the United States and is one of the most widely used software in the world. It mainly includes user interfaces such as ArcMap, ArcCatalog and ArcToolbox. In this study, we mainly use tools such as surface analysis, hydrological analysis, spatial analysis, raster calculation and extraction analysis in ArcMap. Anusplin 4.3 software provides a tool for transparent analysis and interpolation of noisy multivariate data using thin-slab smoothing splines. The software supports this process by providing comprehensive statistical analysis, data diagnostics, and standard errors of spatial distribution. It is usually applied to the interpolation of meteorological factors such as precipitation and temperature, and covariates such as elevation can be introduced for the interpolation of meteorological factors. In this study, rainfall interpolation was mainly performed using Anusplin software. ENVI 5.3 software is a powerful remote sensing image processing software developed by ITT Visual Information Solutions, Inc. The software processing techniques cover image data input/output, image calibration, image enhancement, correction, orthorectification, mosaic, data fusion and various transformations, information extraction, image classification, knowledge-based decision tree classification, integration with GIS, DEM and terrain information extraction, radar data processing, and 3D stereo display analysis. In this study, we mainly use ENVI to extract Modis data, image mosaic, projection, and waveband operation to obtain NDVI data.

The rainfall data were collected from 14 meteorological stations (Taiyuan, Taigu, Yushe, Jiexiu, Linfen, Anze, Changzhi, Xiangyuan, Houma, Yuanqu, Yangcheng, Xinxiang, Mengjin, Zhengzhou) around the Qin River basin. Daily precipitation data were then calculated on a monthly scale. DEM data were obtained using the Advanced Spaceborne Thermal Emission and Reflection Radiometer Global Digital Elevation Model (ASTER GDEM) for August 2018.NDVI data were collected every 16 days based on Landsat remote sensing data and annual average NDVI values were calculated using the maximum synthesis algorithm. The years 1990, 2000 and 2010 for the Chin River basin are NDVI data with Landsat 5 clouds less than 50%; the year 2018 is NDVI data with Landsat 8.

The Chinese government proposed to implement the policy of the Grain for Green project in 1999, choosing 1990 as the starting year of the study and 2010 as the interval point, which can better compare the first ten years and the last ten years of the policy. Due to data acquisition, 2018 is selected as the end year. Relevant data sources are presented in **Table 1**.

**Table 1:** Sources of datasets used in this study.

Data	Source	Spatial resolution	Temporal period
**DEM**	National Tibetan Plateau Data Center (http://data.tpdc.ac.cn)	1000 m	
**Rainfall**	China Meteorological Data Service Centre (https://data.tpdc.ac.cn)	Point	1990–2018
**Soil**	National Tibetan Plateau Data Center (https://data.tpdc.ac.cn)	1000 m	
**Land use**	Resources and Environmental Science and Chinese Academy of Sciences (https://www.resdc.cn)	100 m	1990–2018
**NDVI**	United States Geological Survey (https://earthexplorer.usgs.gov/)	30 m	1990–2018

### 2.3 Methods

**Fig 2:**
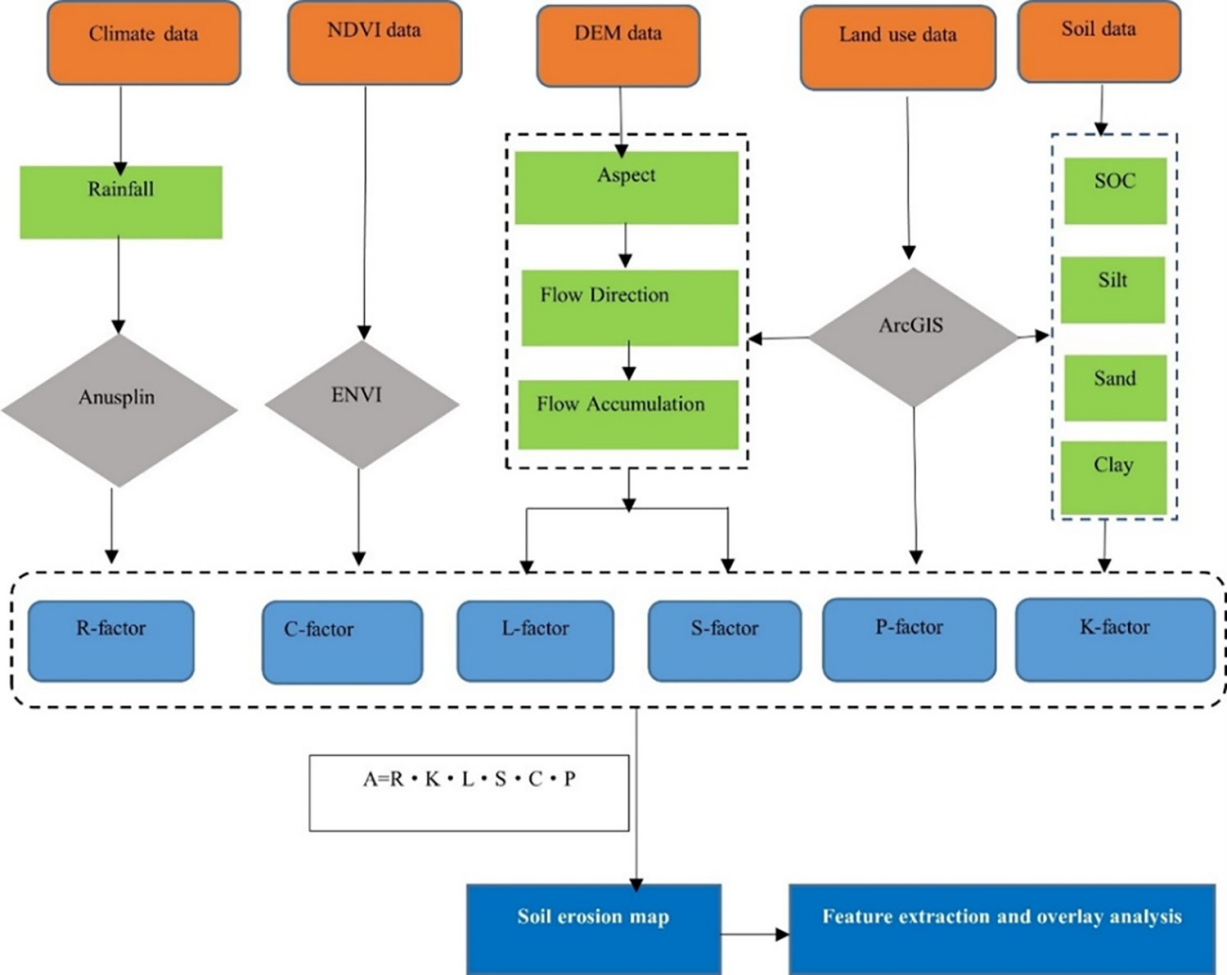
Flow chart of methodology.

RUSLE compensates for the limitations of field observations in large-scale applications, and it is also suitable for multiscale simulation to achieve good results [34–36]. Precipitation erosivity, vegetation cover, soil erodibility, slope length, and water and soil conservation measures are comprehensively considered on RUSLE model [37]. The calculation process for the RUSLE method is shown in Fig 2. The overall formula is as follows:

A=R⋅K⋅LS⋅C⋅P (1)

where *A* indicate the annual average soil erosion in t hm^−2^ a^−1^; *R* indicate the rainfall erosivity factor, and its unit is t ha MJ^−1^ mm^−1^; *K* indicates the soil erodibility factor, and its unit is t ha MJ^−1^ mm^−1^; *L* indicate the slope-length factor (dimensionless); *S* indicate the slope-steepness factor (dimensionless); *C* indicate the land cover and management factor (dimensionless); and *P* indicate the conservation practice factor (dimensionless).

#### 2.3.1 Rainfall erosivity factor (R)

R-factor reflects the potential of rainfall to cause soil erosion. It is an empirical measure of the rainfall erosivity, which is affected by volume, duration, intensity, and the pattern of rainfall, as well as the amount and rate of the resulting overland run off [38,39]. It can be calculated based on the rainfall kinetic energy (E) and the maximum 30-minute rainfall intensity (I_30_) [23]. Eq (2) is suited for investigation of study areas that lack high temporal resolution rainfall records and incorporates monthly and annual rainfall values [40]. This equation was used for estimating monthly rainfall erosivity. Its value is mainly determined by kinetic energy and precipitation. In order to determine the intensity, it is necessary to collect detailed and continuous precipitation data and use the storm rainfall erosivity index (EI_30_) for quantitative calculation [41]. In most areas of China, the data in the 1990s are more difficult to obtain. In order to evaluate the value of R factor, the calculation is based on the monthly average precipitation. The Wischmeier and Smith [23] empirical formula, modifed by Arnoldus [42] was used:

$$R=\sum_{i=1}^{12}1.735\times 10^{1.5\mathrm{lg}(\frac{p_{i}^{2}}{p})-0.08188}$$ (2)

where *p*_*i*_ indicate the monthly precipitation, and its unit is mm; *p* indicate the annual precipitation, and its unit is mm.

According to the daily rainfall data of 13 stations around the study area, we calculated the rainfall erosivity according to Formula (2), and obtained the R-value map (S1 Fig) of the study area from 1990 to 2018 by using Anusplin 4.3 software.

#### 2.3.2 Soil erodibility factor (K)

The soil erodibility is influenced by soil properties, which are determined by soil type. Fine soil is classified into clay, sand, and silt. The definitions of silt and sand differ between the United States and the rest of the world. Silt and sand are defined internationally and by the United States as 0.002–0.02 and 0.02–2, and as 0.002–0.05 and 0.05–2 mm, respectively. Clay is referred to as particles with a size of < 0.002 mm.

The calculation method of K value is mainly related to sand, powder, clay and organic matter content. Erosion Productivity Impact Calculator (EPIC) model is more focused on soil properties and can reflect soil erodibility in a more scientific way [43,44]. The K factor was estimated using EPIC model and has been expressed as follows:

$$K=\{0.2+0.3*\exp[-0.0256*SAN*(1.0-\frac{SIL}{100})]\}\times {\left(\frac{SIL}{CLA+SIL}\right)}^{0.3}\times \{1.0-\frac{0.25*C}{C+\exp(3.72-2.95*C)}\}\times (1.0‐\frac{0.7*S_{n}}{S_{n}+\exp(22.9S_{n}-5.51)})\times 0.1317$$ (3)

where *CLA*, *SIL*, and *SAN* represents the contents (%) of clay, silt, and sand in the soil, respectively; *C* represents the soil organic carbon content (%), and Sn = 1 − *SAN* / 100. Since the formula was proposed by the United States for particle size and sand size, multiplying it by 0.1317 converts the United States units into international units (t ha h ha^−1^ MJ^−1^ mm^−1^). Soil organic matter contains a lot of organic carbon, and its content (C value) can be obtained by dividing the content of organic matter by 1.724.

The K factor reflects the difference in the amount of erosion caused by the different properties of the soil itself, and the soil texture, organic matter content, soil structure, and permeability determine the erodibility of the soil. In this study, the soil particle composition data and soil organic carbon data were used, and the soil erodibility K value map was obtained by Formula 3 (S2 Fig).

#### 2.3.3 Slope-length (L) and steepness (S) factors

Regional topography can accelerate erosion caused by rainfall. Slope runoff is the driving force of soil erosion and sediment transport. Slope length determines the variation of flow energy and the transport mechanics of flow and sediment. It is an important factor affecting slope erosion [45]. The amount of erosion from uphill to downhill increases with the increasing of slope length, but the relationship between them is not conclusive [46]. Some studies show that the erosion decreases with the increase of slope length, which may be related to the flow and slope [47]. The amount of erosion fluctuates with the increase of slope length. When the slope runoff is the same, there is a critical slope length value at which the amount of erosion reaches the maximum. However, the current research finding showed that there is no final conclusion on this critical value [48]. Therefore, the calculation of L-factor was carried out using the classical formula proposed by Wischmeier and Smith [23] as follows:

$$L={\left(\frac{\lambda }{22.13}\right)}^{\alpha }$$ (4)

λ=Flowaccumulation*Cellsize (5)

$$\alpha =\frac{\beta }{\beta +1}$$ (6)

$$\beta =\frac{\sin\theta /0.0896}{3.0\times {\left(\sin\theta \right)}^{0.8}+0.56}$$ (7)

where *λ* indicate the slope length in m; Flow accumulation indicate Cumulative uphill area of unit; Cellsize is the spatial resolution of the grid; α indicate the slope-length index; *β* indicate the erosion ratio of rill to inter-rill erosion; *θ* indicate the slope value extracted from DEM in degree [°], and 22.13 m indicate the standard slope length.

S represents the effect of the slope gradient on soil erosion phenomena. It is described as the ratio of soil loss from a field of existing slope condition to that from a field of 9% slope with all other conditions being identical. Since slopes≥10° comprise about 48% of the entire study area, we used the formula of McCool et al. to calculate the slope factor in the areas with a slope of < 10° [49], and Liu et al improved formula was used to calculate the slope factor in areas with a slope of > 10° [24]. The calculation formula is as follows:

$$\left\{ \begin{array}{l} \;10.8*sin\theta +0.03\;\theta <5{}^{\circ}\\ S=16.8*sin\theta ‐0.50\;5{}^{\circ}\leq \theta <10{}^{\circ}\\ \;21.9*sin\theta ‐0.96\;\theta \geq 10{}^{\circ} \end{array}\right.$$ (8)

where, θ represents the slope value (°).

The DEM data with a resolution of 30 m in the study area are processed with depression filling, flow direction, grid calculation, etc., and the slope is extracted θ,And slope length λ Value, use Formulas (4 ~ 8) to calculate the value of L and s, and get the distribution map of LS value in ArcGIS software (S3 Fig).

#### 2.3.4 Cover management factor (C)

The degree of soil and water loss inhibition is related to vegetation cover through field management measures, which is represented by the value of C. NDVI can used to represent the surface-vegetation coverage, which can comprehensively reflect the form, type, and growth status of vegetation coverage in a pixel [50,51]. Therefore, the C factor is estimated using the NDVI [52], and the formula is as follows:

$$C=\exp(-2.5\times \frac{NDVI}{1-NDVI})$$ (9)

where *C* represents the cover management factor; *NDVI* represents the normalized vegetation index, and its range is 0−1.

The C value of each pixel unit is calculated byArcGIS10.2 and Formula (9), and the distribution map of C value in the study area in 1990,2000, 2010 and 2018 is obtained (S4 Fig).

#### 2.3.5 Conservation practice factor (P)

P is used to explain the inhibitory effects of water and soil conservation measures on soil erosion. The P factor has a range of 0−1. When P = 0, there is no soil erosion after the water and soil conservation measures being implemented. When P = 1, it implies that no water and soil conservation measures were taken in that area. At present, there is no universal P-factor assignment standard in large-scale studies to objectively reflect the spatial distribution pattern of the factors of water and soil conservation measures. According to previous studies [53–56], the P factor was assigned based on land use, and the results are shown in (Table 2**)**. According to the land use types in the study area, combined with Table 2, this paper uses the reclassification tool of ArcGIS10.2 to obtain the spatial distribution map of P value (S5 Fig)

**Table 2:** Assignment of P values based on land use.

Level 1 number	First-level land-use classification	Level 2 number	Secondary land-use classification	P Value
**1**	Cultivated land	11	Paddy field	0.01
		12	Dry land	0.4
**2**	Woodland	21	Forestland	1
		22	Shrub woodland	1
		23	Sparse woodland	1
		24	Other woodlands	0.7
**3**	Grassland	31	High coverage grassland	1
		32	Moderate coverage grassland	1
		33	Low coverage grassland	1
**4**	Water area	41	Canal	0
		42	Lake	0
		43	Reservoir pond	0
		44	Permanent glacier and snow	1
		45	Tidal flat	0.2
		46	Beach land	0.01
**5**	Urban and rural, industrial and mining, residential land	51	Urban land	0
		52	Rural Settlement	0
		53	Other construction land	0
**6**	Unused land	61	Sand	1
		62	Gobi	1
		63	Saline alkali land	1
		64	Marshland	0.7
		65	Bare land	1
		66	Bare rock land	1
		67	Other land	1

## 3 Results

### 3.1 Spatiotemporal variation of the soil erosion degree

**Fig 3:**
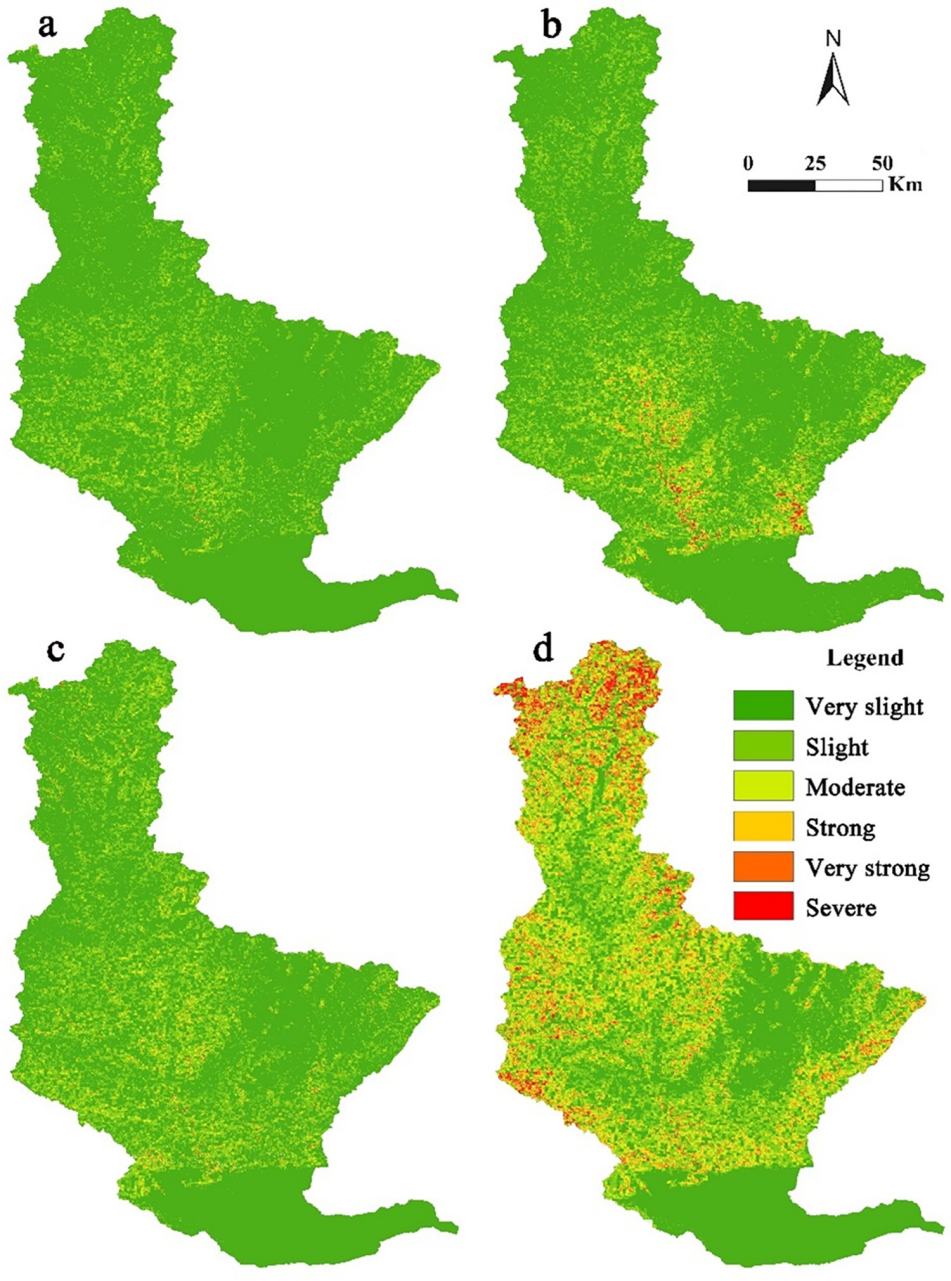
Soil erosion in 1990 (a); soil erosion in 2000 (b); soil erosion in 2010 (c); soil erosion in 2018 (d). According to Formula 1, we multiply the six factors R, K, L, S, C and P in ArcGIS 10.2 software to obtain soil erosion maps in 1990, 2000, 2010 and 2018. The scope of the Qin River Basin was determined based on DEM and eight-direction (D8) algorithm. Based on authors’ field investigation, we have modified and adjusted the vector boundary.

We calculated soil erosion area (Table 3) and prepared the spatial distribution map (Fig 3) of the Qin River Basin based on the standard for classification and gradation of soil erosion (SL 190–2007) issued by the Ministry of Water Resources, China. Which is divided into six levels. The specific classification standard is shown in table (S1 Table). For details, please refer to the Department of international cooperation and science and technology of the Ministry of water resources of the people’s Republic of China (http://gjkj.mwr.gov.cn/).

**Table 3:** Soil erosion severity class areal distribution from 1990 to 2018.

Classes	1990	2000	2010	2018
Very slight	83.14	72.79	71.22	39.60
Slight	15.28	21.30	24.31	28.64
Moderate	1.32	3.62	3.27	15.47
Strong	0.19	1.27	0.79	8.27
Very Strong	0.05	0.71	0.33	6.03
Severe	0.02	0.30	0.08	1.99

The average soil erosion of the Qin River Basin in 1990, 2000, 2010, and 2018 were 3.04, 6.73, 5.65, and 35.05 t hm^−2^ a^−1^, respectively. Of these, 1990 in general had very slight erosion, 2000 and 2010 slight erosion, and 2018 moderate erosion. The Qin River Basin suffered the most serious erosion in 2018, because it had the highest rainfall among all years. The greater the rainfall, the more serious the soil erosion.

Table 3 shows that from 1990 to 2018, the Qin River Basin was dominated by very slight erosion. From 1990 to 2010, very slight soil erosion accounted for more than 70% of the area, which was 39.6% in 2018. However, from 1990 to 2018, the proportion of areas with very slight soil erosion decreased by 43.54%, indicating that soil erosion intensity in this study area demonstrated an increasing trend.

As shown in Fig 3, the areas severely eroded are distributed in the middle and upper reaches of the basin. The upper reaches of the study area are mountainous, while the middle reaches are hilly. The mountain and hilly regions have steep slopes, and the destruction of vegetation is serious in these regions. In contrast, soil erosion is light in the plain region because of the lower slope and minor changes in the land-use pattern.

### 3.2. Analysis of changes in soil erosion under different land-use types

**Fig 4:**
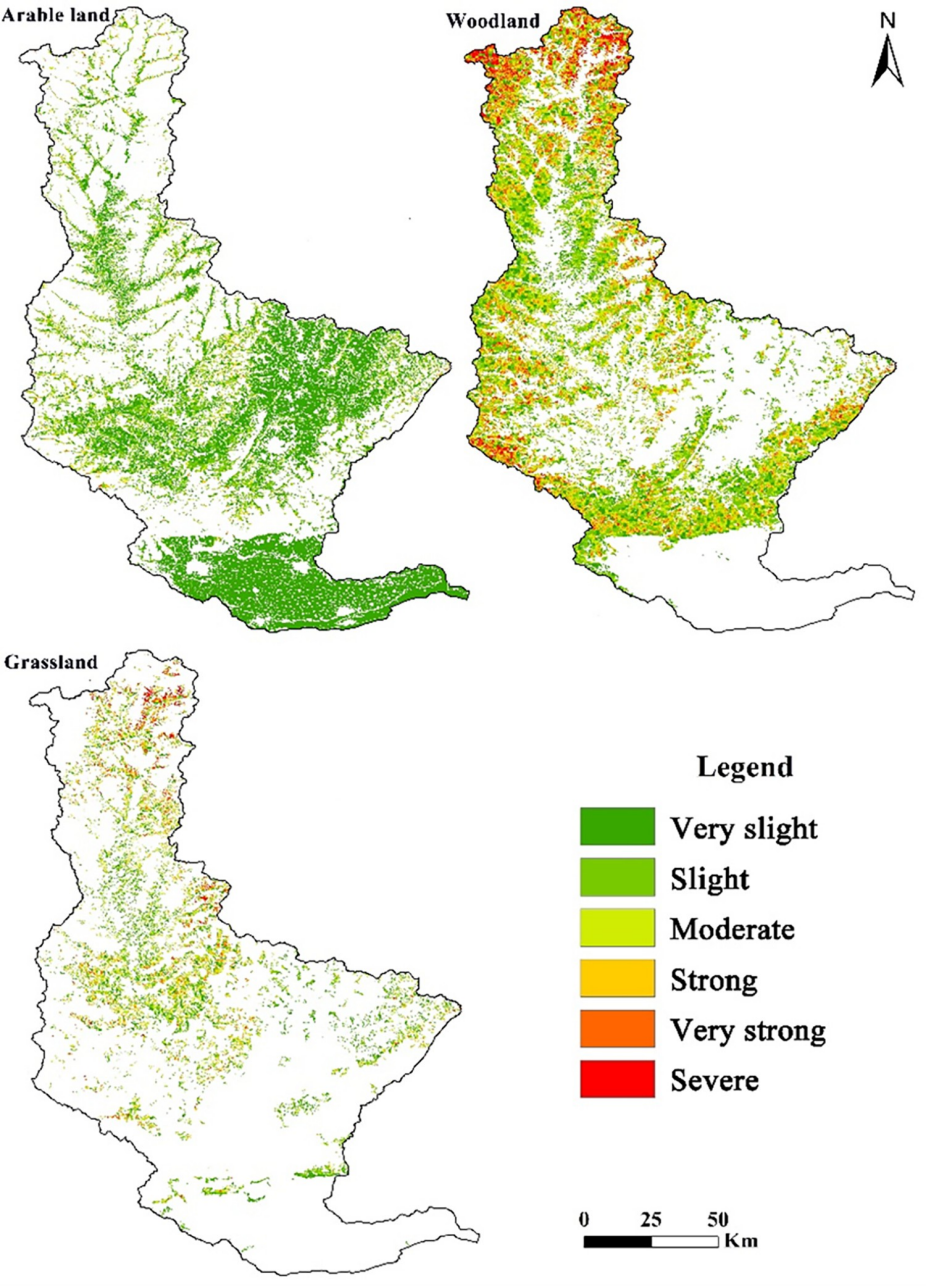
Distribution of the soil-erosion intensity of different land-use types for Qin River Basin. We used ArcGIS software to extract the areas of cultivated land, forest land and grassland, and superimposed soil erosion with the extracted areas to obtain the distribution map of soil erosion intensity for different land uses. The scope of the Qin River Basin was determined based on DEM and eight-direction (D8) algorithm. Based on authors’ field investigation, we have modified and adjusted the vector boundary.

As a form of human activities, land use has the most direct impact on soil erosion. Different land-use types showed significant differences in soil erosion. Nineteen level II land use types were used to evaluate their specific soil erosion characteristics. The results are listed in (Table 4). Based on previous research results and P values in Table 2, the amount of soil erosion of five land use types, including canals, reservoirs, ponds, urban land, rural residential areas, and other construction land is 0. These five types of land use did not undergo soil erosion and hence were excluded from further analysis. As the unused land area in the Qin River Basin is very little, only the spatial distribution of the intensities of soil erosion of cultivated land, forestland, and grassland were researched, and their spatial distribution are shown in Fig 4.

**Table 4:** Spatiotemporal changes in soil erosion for land-use patterns from 1990 to 2018.

	1990			2000			2010			2018		
Land-use type	Area ratio (%)	Soil erosion (t hm^−2^ a^−1^)	Total soil erosion (10^4^ t)	Area ratio (%)	Soil erosion (t hm^−2^ a^−1^)	Total soil erosion (104 t)	Area ratio (%)	Soil erosion (t hm^−2^ a^−1^)	Total soil erosion (10^4^ t)	Area ratio (%)	Soil erosion (t hm^−2^ a^−1^)	Total soil erosion (10^4^ t)
**Paddy field**										0.01	0.02	0.00
**Dry land**	37.82	1.69	101.02	40.03	3.40	200.45	39.70	3.01	176.56	37.52	6.89	381.46
**Forested land**	31.48	2.98	148.47	33.57	7.82	386.33	33.59	6.48	321.12	34.23	46.26	2337.14
**Shrub woodland**	1.63	4.62	11.95	1.76	14.97	38.84	1.77	8.26	21.53	1.71	26.68	67.32
**Sparse woodland**	6.84	6.80	73.62	7.22	15.18	161.40	7.19	10.88	115.49	6.97	33.07	340.41
**Other woodlands**	6.84	1.17	0.25	0.31	4.38	2.00	0.23	3.93	1.32	0.22	22.39	7.43
**High coverage grassland**	11.24	6.23	110.78	12.28	10.47	189.17	12.24	10.69	193.03	11.87	37.21	652.15
**Moderate coverage grassland**	0.29	5.89	2.74	0.32	23.73	11.05	0.21	11.54	3.65	0.19	20.42	5.85
**Low coverage grassland**	0.10	2.02	0.32	0.11	9.77	1.58	0.08	3.75	0.42	0.09	11.77	1.51
**Canal**	0.32	0.00	0.00	0.35	0.00	0.00	0.40	0.00	0.00	0.38	0.00	0.00
**Reservoir pond**	0.06	0.00	0.00	0.07	0.00	0.00	0.12	0.00	0.00	0.15	0.00	0.00
**Beach land**	0.23	0.14	0.05	0.24	0.48	0.17	0.17	0.06	0.01	0.16	0.08	0.02
**Urban land**	0.31	0.00	0.00	0.65	0.00	0.00	0.92	0.00	0.00	1.37	0.00	0.00
**Rural Settlement**	2.71	0.00	0.00	2.94	0.00	0.00	2.98	0.00	0.00	4.04	0.00	0.00
**Other construction land**	0.10	0.00	0.00	0.13	0.00	0.00	0.39	0.00	0.00	1.07	0.00	0.00
**Sand**	0.00	0.05	0.00	0.01	2.69	0.03						
**Gobi**										0.00	19.82	0.02
**Bare land**							0.01	12.46	0.15	0.01	11.77	0.22
**Bare rock land**	0.00	3.98	0.03	0.01	24.93	0.19	0.01	3.66	0.03	0.01	4.13	0.03

Note: t hm^−2^ a^−1^ indicate soil erosion unit; t indicate ton; hm^-2^ indicate hectare; a indicate year.

As shown in Table 4, the three main land types in the Qin River Basin are dryland, forested land, and high coverage grassland, which account for more than 80% of the total area. Dryland accounts for more than 37% of the total area, and forestland accounts for more than 31% of the total area. In 1990, the strongest soil erosion occurred in sparse woodland. From 2000 to 2010, the strongest soil erosion occurred in moderate coverage grassland. High and Moderate coverage grassland were moderately eroded. In 2018, the land with the strongest soil erosion was forested land, followed by high-coverage grassland and sparse woodland, both of which exhibited moderate erosion. From 1990 to 2018, the amount of soil erosion in the forestland was the highest, and it showed an increasing trend, especially in 2018, when it amounted to (2337.14 × 10^4^ t) in ton. Furthermore, during this period, the loss of high-coverage grassland was higher. For beach land, the degree and amount of erosion were the lowest with a decreasing trend. Qin River Basin is mainly rocky mountains and hilly region of earth and rock with steep slopes. In addition, the area of forestland, shrub woodland, sparse woodland and high coverage grassland account for a large proportion. Therefore, the amount of soil erosion in these areas is relatively large.

Fig 4 and Table 5 show that the study area mainly composed of cultivated land and forestland. Based on land-use types, there are important differences in the distribution of soil erosion intensity for this study area. Arable land exhibited mostly very slight erosion occupying an area of 64.39%, while severe erosion accounted only for 0.07% of the area. Woodlands and grasslands were predominantly slightly and moderately eroded, respectively. Soil-erosion intensity of woodland and grassland increased and then decreased, and slight accounting for the highest proportion. Woodland showed strong soil erosion in 29.06% of the area, and the protection of forest soil erosion should be strengthened.

**Table 5:** Soil erosion proportions of different land-use types in the Qin River Basin (%).

Classes	Arable land	Woodland	Grassland
**Very slight**	64.39	13.83	20.22
**Slight**	27.44	32.43	34.17
**Moderate**	5.90	24.68	21.49
**Strong**	1.58	14.37	12.36
**Very Strong**	0.62	10.88	9.12
**Severe**	0.07	3.81	2.64

### 3.3. Variations in soil erosion with slope

**Fig 5:**
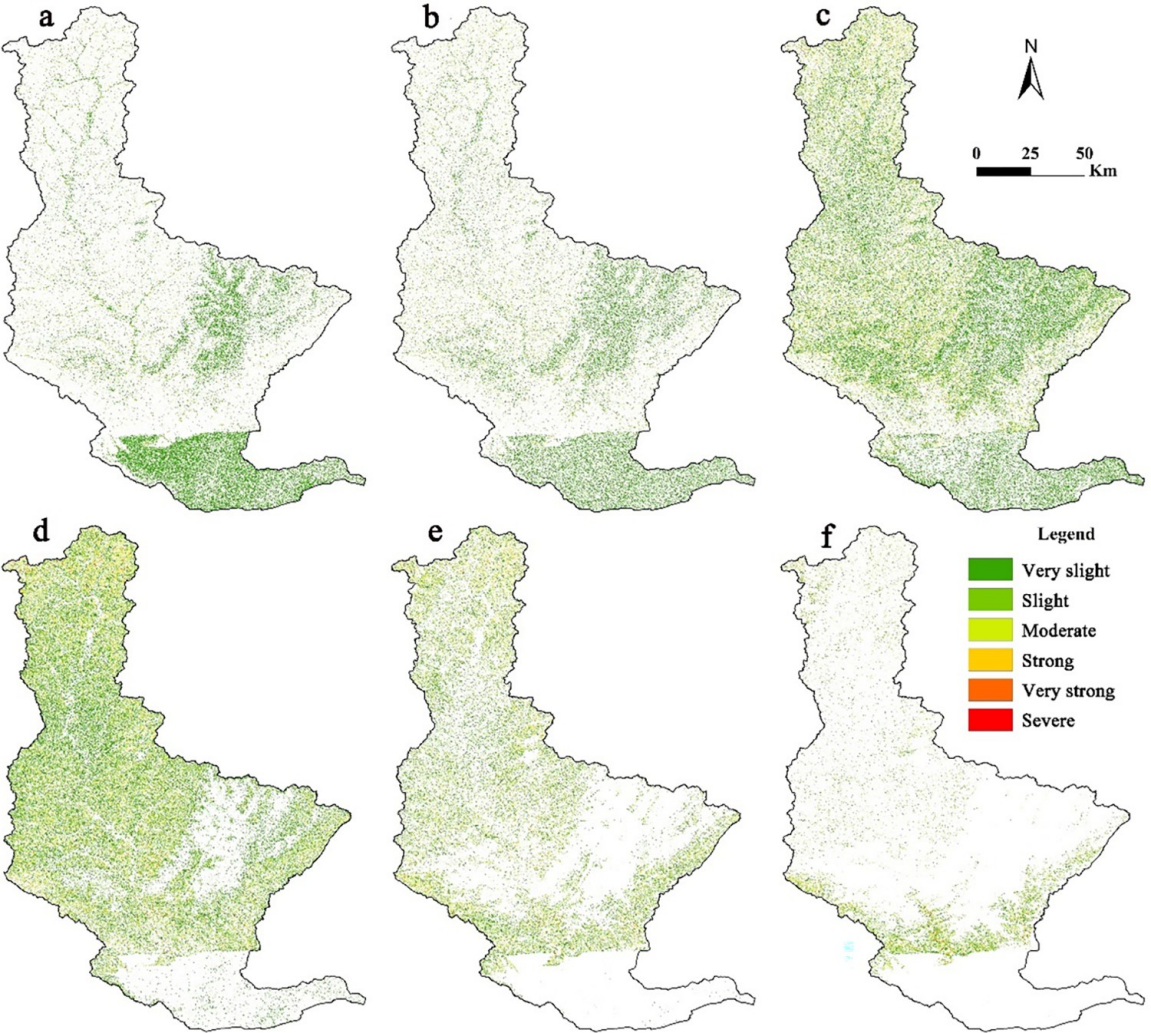
Distribution of the intensity of soil erosion at regions with varying slopes in the Qin River Basin. Slop≤5°(a); 5°<Slop<8°(b); 8°≤Slop≤15°(c); 15°≤Slop<25°(d); 25°≤Slop<35°(e); Slop≥35°(f). Based on the superposition analysis function of the ArcGIS software, we extracted the slopes of the Qin River Basin, and divided them into 6 categories according to the gradient classification standards. The six types of slope maps and soil erosion maps were masked to obtain soil erosion classification maps of different slopes. The scope of the Qin River Basin was determined based on DEM and eight-direction (D8) algorithm. Based on authors’ field investigation, we have modified and adjusted the vector boundary.

A significant topographic factor affecting soil erosion is slope. In this study, the extracted slope was superimposed with the layer of soil erosion intensity to calculate the areas of soil erosion under different slopes. The results are shown in Tables 6 and **7** and Fig 5.

**Table 6:** Soil erosion intensity under different slopes.

Slope	Area ratio (%)	Soil erosion (t hm^−2^ a^−1^)	Total soil erosion (10^4^ t)	Proportion of volume in total soil erosion (%)
**≤ 5°**	12.76	4.09	76.82	5.09
**5~8°**	12.40	5.55	101.32	6.71
**8~15°**	27.27	8.73	350.40	23.21
**15~25°**	29.09	12.69	543.13	35.97
**25~35°**	13.76	15.29	309.44	20.49
**≥ 35°**	4.73	18.50	128.73	8.53

Note: Slope Classification based on the standard for classification and gradation of soil erosion (SL 190–2007) issued by the Ministry of Water Resources, China.

**Table 7:** Areas of varying intensities of soil erosion based on slope (%).

Classes	≤ 5°	5~8°	8~15°	15~25°	25~35°	≥ 35°
**Very slight**	80.01	72.08	56.09	38.24	30.32	25.84
**Slight**	16.17	22.54	34.88	47.22	51.00	50.86
**Moderate**	2.90	4.18	7.10	11.33	14.27	16.65
**Strong**	0.65	0.87	1.47	2.47	3.41	4.41
**Very Strong**	0.24	0.29	0.41	0.66	0.87	1.71
**`Severe**	0.04	0.05	0.05	0.08	0.12	0.52

Areas with a slope of > 15° account for approximately 47% of the basin area. Table 6 indicates that as the slope increases, soil erosion increases. Furthermore, the soil erosion difference between slope grades varies from 1.46–3.96 t hm^−2^ a^−1^. Areas with a slope of ≤ 5° show very slight erosion, and those with a slope of > 5° are slightly eroded. The highest total soil erosion percentage occurs in areas that have a slope of 15~25°, accounting for 35.97% of the area of total soil erosion. Areas with this range of slope are thus most important for preventing and controlling soil erosion. It is suggested that the rational utilization of land resources, control of soil and water loss, return of farmland to forest, and terrace planting should be strengthened to mitigate soil erosion.

Fig 5 and Table 7 show that there are prominent differences in the distribution of the erosion intensity with varying slopes. In general, the Qin River Basin is dominated by very slight and slight erosion. With the increase in slope, the proportion of very slight erosion decreases. Very slight erosion was as high as 80% for 0–5°, and it decreased to 25.84% for ≥ 35°. Other erosion categories increased with the increase in slope, evidenced by their proportions being increased. This confirms that slope has an important influence on soil erosion.

### 3.4. Changes in soil erosion at different altitudes

**Fig 6:**
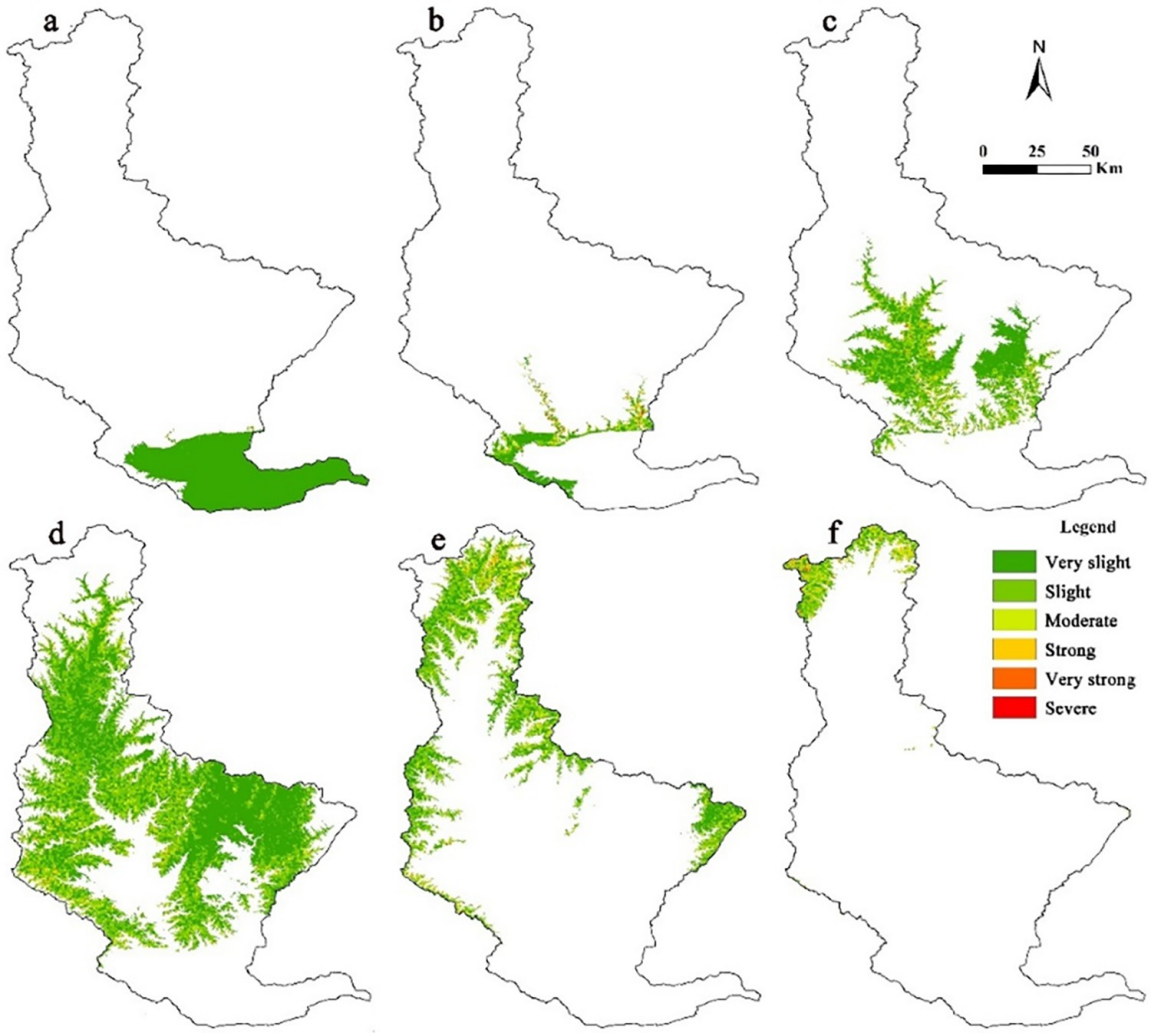
Distribution of the soil-erosion intensity at different altitudes. Altitude≤200m(a); 200m<Altitude≤500m(b); 500m<Altitude≤800m(c); 800m<Altitude≤1200m(d); 1200m<Altitude≤1500m(e); Altitude>1500m(f). According to reference 62, we divided the Qin River Basin into six grades, using the spatial analysis function of ArcGIS software to extract the area at different altitudes, and using the mask extraction function to obtain the soil erosion degree maps at different altitudes. The scope of the Qin River Basin was determined based on DEM and eight-direction (D8) algorithm. Based on authors’ field investigation, we have modified and adjusted the vector boundary.

Six altitude ranges were identified using watershed DEM. For different altitudes in the watershed, these altitude ranges were superimposed with the soil-erosion grid layer to obtain the amount of soil erosion of different altitude ranges (Tables 8 and 9 and Fig 6).

**Table 8:** Soil erosion at different altitudes.

Altitude	Area ratio (%)	Soil erosion (t hm^−2^ a^−1^)	Total soil erosion (10^4^ t)	Proportion of volume in total soil erosion (%)
**≤ 200 m**	11.92	0.40	7.10	0.47
**200~500 m**	3.47	15.23	77.78	5.15
**500~800 m**	14.95	10.71	237.66	15.74
**800~1200 m**	48.03	9.39	666.97	44.18
**1200~1500 m**	17.94	14.99	389.71	25.81
**> 1500 m**	3.70	24.97	130.61	8.65

Note: Altitude range class standard reference [64].’

**Table 9:** Proportion of areas (%) with varying intensities of soil erosion at different altitude ranges.

Classes	≤ 200 m	200~500 m	500~800 m	800~1200 m	1200~1500 m	> 1500 m
**Very slight**	99.41	54.61	49.42	49.46	30.30	17.67
**Slight**	0.44	28.71	39.09	41.39	50.91	43.39
**Moderate**	0.08	9.67	8.45	7.57	14.86	26.58
**Strong**	0.04	3.66	2.12	1.27	3.19	9.47
**Very Strong**	0.03	2.20	0.80	0.29	0.69	2.60
**Severe**	0.01	1.16	0.11	0.02	0.05	0.29

Table 8 show that intensity of soil erosion in the Qin River Basin varies significantly with altitude. Soil erosion for an altitude of < 200 m is only 0.4 t hm^−2^ a^−1^, which is negligible. Areas that lie in the altitude range of 200–1500 m are slightly eroded. The areas that are > 1500 m show a moderate erosion rate of 24.97 t hm^−2^ a^−1^. Altitude range of 500–1500 m is where soil erosion mainly occurs, accounting for 85.73% of the total soil erosion volume.

Soil erosion at different altitudes (Fig 6 and Table 9) in the Qin River Basin was slight and moderate. Significant differences in soil erosion were observed at different altitudes range. The soil-erosion degree for areas with an altitude of < 1200 m demonstrated that the degree of soil erosion is mainly very slight and slight. The categories of very slight to slight soil erosion show an increasing trend in the regions with an altitude of > 1200 m, while moderate erosion shows a decreasing trend. With the increase in altitude, the proportion of very slight soil erosion tends to decrease, and the other categories tend to increase. Therefore, attention should be paid to soil erosion at high altitudes to reduce water and soil loss by increasing the vegetation and ground roughness.

## 4 Discussion

### 4.1 Spatial characteristics of soil erosion in different geographical elements

Soil erosion has a serious impact on habitat and productivity [57]. The total erosion in the Qin River Basin from 1990 to 2018 was 1509.84 × 10^4^ t·a^−1^, of which soil erosion was 10.26 t hm^−2^ a^−1^. Overall the soil erosion in the study area categorized as very slight and slight. Very slight erosion accounted for 51.15% of the study area. Gao et al. studied the Loess Plateau and found that the soil erosion is 50.48% [58], which is consistent with the results of this research. The spatial pattern characteristics of the soil-erosion intensity in this study area were assessed. Compared to the results of previous research [59], the soil-erosion intensity in mountainous and hilly areas is significantly higher than that in plain areas. This is because the Qin River Basin is a typical transition area of "mountain-hill-plain", and the mountain and hilly areas have steep slopes, higher elevations and more serious erosion. Therefore, this study is unique and significant from the point of soil erosion mitigation.

The intensities of soil erosion can be distinguished and evaluated by analyzing soil erosion patterns through various environmental factors, such as, land use, slope, and altitude. Sun et al obtained similar results for the Loess Plateau [60]. He et al. studied soil erosion with respect to varying land uses in the Qi River Basin of the Taihang Mountain and found that cultivated land was very slightly eroded, and soil erosion occurred in 71.45% of the total area. Furthermore, they found that soil erosion of woodland and grassland was slight, accounting for 35.52% and 33.23% of the area, respectively [61]. This is consistent with the areas that experienced slight erosion of arable land (64.38%), woodland (32.42%), and grassland (34.17%) of this study. Soil erosion is more serious in the forested and grassland areas of the Qin River Basin, due to the fact that grassland and forested land are mostly distributed in high-elevation areas with large topographic relief, where the potential soil erosion is larger and the soil erosion intensity is higher. Among the secondary land types, soil erosion is more serious in sparse forest land and forested land; soil erosion is more serious in high cover grassland and medium cover grassland. Therefore, the specific land use types can be adjusted in the Qin River Basin in terms of erosion control and land use management, and the area share of other woodlands, shrublands and low-cover grasslands can be increased.

Zhang et al. suggested that effect of slope on soil erosion is unique rather than displaying a proportional relationship [30]. Rao et al revealed that areas with a slope of 15–25° were the most severely eroded in China [62]. Similarly, the soil-erosion degree in this study area increased as the slope increased and the degree of soil erosion increased. A slope limit of 15° affects soil erosion. The areas with a slope of > 15° accounted for 47.57%, while the proportion of soil erosion in such areas was 64.99%. These results are similar with the results of Jiang et al. in the Jinsha River [63]. Wang et al. showed that the critical values of soil erosion are different for different geomorphic patterns [64]. With an increase in altitude and gradient, soil erosion first increases and then decreases, and the critical range of the most serious erosion varies for different research scales and locations. Rao et al. found that, in China, areas with an altitude of 1000–2000 m are of serious concern in terms of soil erosion. This is similar with the concern areas consisting of regions with an altitude of > 1200 m [62]. The greater the slope and undulation, the greater the instability of the soil on the slope, and the greater the possibility of downward movement under the action of external forces. Therefore, the Qin River basin should focus on reasonable adjustment of land use types in the areas of 15°~25° and above 1200~1500m, and enhance soil and water conservation capacity by returning farmland to forest and grass.

### 4.2 Major finding and applicability of this study

This study found that precipitation has a greater effect on soil erosion, and the greater the erosive power of rainfall, the more severe the soil erosion (S1 Fig). Because precipitation is very closely related to the magnitude of soil erosion on slopes, it can increase erosion by increasing runoff and thus erosion, making erosion increase with increasing precipitation [32]. In the mountainous areas of the Qin River basin erosion is dominated by raindrop splash erosion, sheet flow and fine gully erosion, and precipitation exceeds the effect of slope and elevation on soil erosion [64]; within the Qin River basin, erosion intensity is mainly the result of the combined effect of precipitation, land use, slope and elevation. Since the terrain factor is basically unchanged in a short period of time, the interference of human activities on the surface should be adjusted in real time, the adaptive vegetation types should be selected, the natural spatial pattern of vegetation should be optimized, the vegetation cover should be increased, the land use structure should be adjusted according to the slope and elevation, and the zoning management should be improved in key areas to reduce soil erosion and connectivity so as to improve the soil conservation effectiveness.

### 4.3 Limitations and needs of this study

Although many scholars have adopted different methods to effectively reveal the types, mechanisms, spatiotemporal distribution, and dynamic evolution processes of soil erosion at different scales, it is difficult to verify these results [65,66]. The RUSLE model has a simple structure and considers the factors that affect soil erosion with minimum data and calculation requirements for simulation at a large scale [67–69]. Although the 30m resolution data was used for NDVI, the NDVI value in 2018 was low due to the influence of climate environment and cloud cover, which reduces the precision of the simulation results of the RUSLE model to a certain extent. In addition, owing to geographical conditions, the meteorological stations in the study area are few, and only one meteorological station is located in the upstream area. Although rainfall data were obtained from 14 meteorological stations in the surrounding area, they were all scattered. The weather stations in the west and south of China are even more remote and cannot fully reflect the continuous spatial distribution of precipitation. In addition, precipitation is significantly affected by changes in altitude [65], which reduces the reliability of the grid-level precipitation values interpolated, which in turn affects the accuracy of soil-erosion estimation and these could be considered as a limitation of this study. Therefore, it is necessary to improve the accuracy of data on factors including land use, NDVI, and rainfall in future studies to accurately estimate soil erosion.

## 5 Conclusions

Soil erosion poses a major environmental threat to the global terrestrial ecosystem, causing land degradation, affecting agricultural production and food security, and threatening human health and security. The Qin River Basin is a typical mountainous hilly plain area with large topographic undulations and slopes and rich precipitation. It has more serious soil erosion.

We estimate soil-erosion intensity and their temporal distribution characteristics in the Qin River Basin from 1990 to 2018. We found that the soil-erosion intensity showed an increasing trend from 1990 to 2018. Soil erosion of Qin River Basin is closely related to geographical environment factors such as rainfall, land use, slope, and altitude. Woodland, arable land, and grassland are main land patterns in the Qin River Basin. Woodland and grassland experienced slight and moderate erosion, and the arable land showed very slight erosion. Sparse woodland and forestland showed the strongest soil erosion, with the highest occurring in forestland with an increasing trend. The highest soil-erosion intensity happened to the slope range of 15~25° resulting in a weak conservation of soil and water ability. The highest intensity of soil erosion occurs at an altitude range of 1200~1500 m, which is the zone of focus for the protection of soil erosion in the Qin River Basin. These regions need to be delineated as development boundaries and overdevelopment in the area should be strictly controlled. The results of the study also provide a reference for other areas in the world with similar topographic landscapes.

## Supporting information

S1 Fig Distribution of R value in the study area from1990 to 2018. The scope of the Qin River Basin was determined based on DEM and eight-direction (D8) algorithm. Based on authors’ field investigation, we have modified and adjusted the vector boundary.
(TIF)

S2 Fig Distribution of K value in the study area. The scope of the Qin River Basin was determined based on DEM and eight-direction (D8) algorithm. Based on authors’ field investigation, we have modified and adjusted the vector boundary.
(TIF)

S3 Fig Distribution of LS value in the study area. The scope of the Qin River Basin was determined based on DEM and eight-direction (D8) algorithm. Based on authors’ field investigation, we have modified and adjusted the vector boundary.
(TIF)

S4 Fig Distribution of C value in the study area from1990 to 2018. The scope of the Qin River Basin was determined based on DEM and eight-direction (D8) algorithm. Based on authors’ field investigation, we have modified and adjusted the vector boundary.
(TIF)

S5 Fig Distribution of P value in the study area from1990 to 2018. The scope of the Qin River Basin was determined based on DEM and eight-direction (D8) algorithm. Based on authors’ field investigation, we have modified and adjusted the vector boundary.
(TIF)

S1 Table The standard for classification and gradation of soil erosion. (DOCX)
