# Supplementary figures and images for: Spatiotemporal characteristics of soil erosion in a typical watershed consisting of different landscape: A case study of the Qin River Basin
Source: PLoS One. 2022 Oct 3;17(10):e0275470. doi: 10.1371/journal.pone.0275470 (PMC9529098; doi:10.1371/journal.pone.0275470)

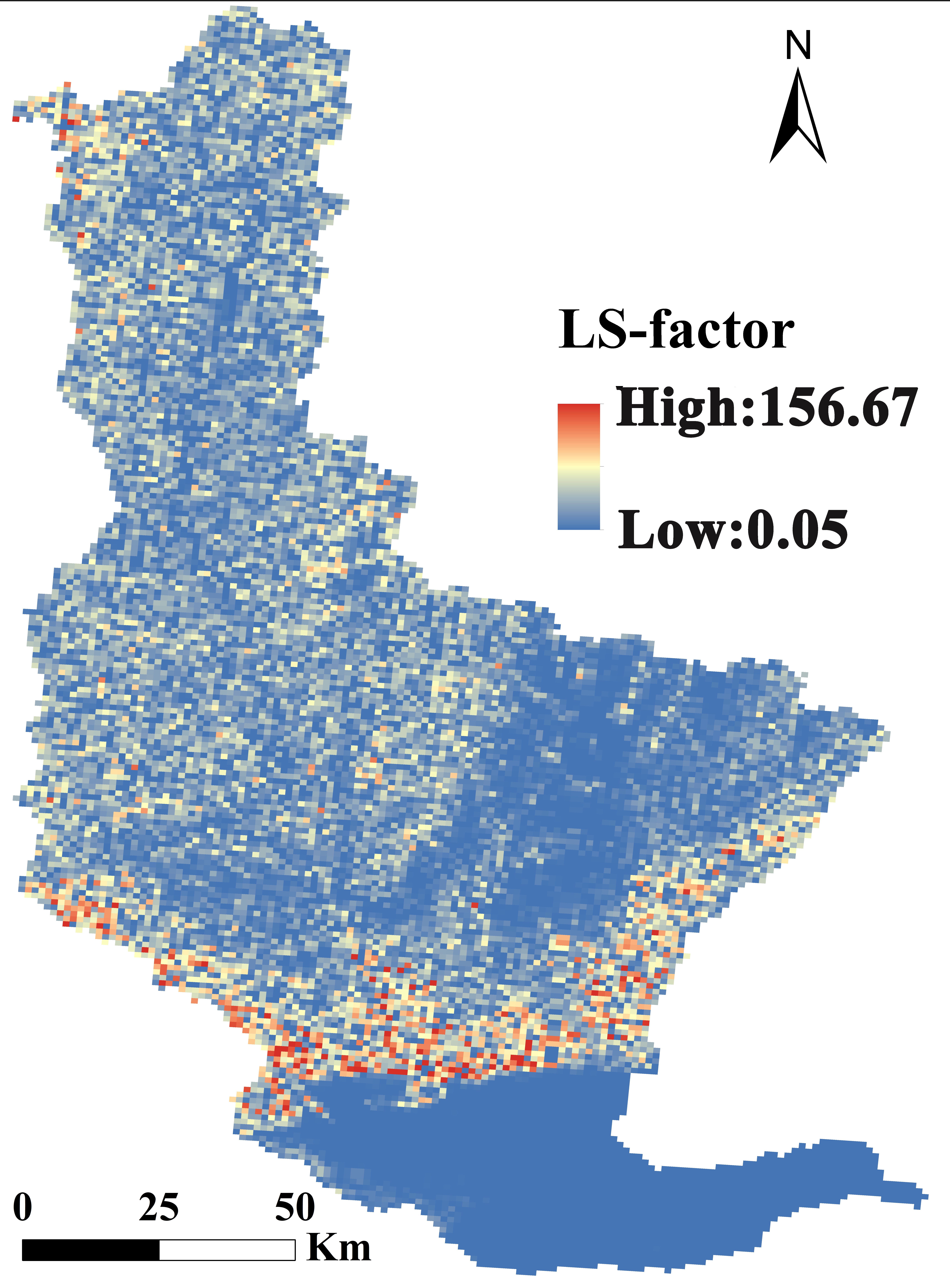

Supplement: S3 Fig — The scope of the Qin River Basin was determined based on DEM and eight-direction (D8) algorithm. Based on authors’ field investigation, we have modified and adjusted the vector boundary. (TIF) [file pone.0275470.s003.tif]
