# Supplementary material for: Spatiotemporal characteristics of soil erosion in a typical watershed consisting of different landscape: A case study of the Qin River Basin
Source: PLoS One. 2022 Oct 3;17(10):e0275470. doi: 10.1371/journal.pone.0275470 (PMC9529098; doi:10.1371/journal.pone.0275470)
Supplement: S1 Table — (DOCX) [file pone.0275470.s006.docx]

**S6 Table.** **The standard for classification and gradation of soil erosion.**

| Serial number | Reference values | Soil erosion class |
| --- | --- | --- |
| 1 | ＜5 | very slight |
| 2 | 5≤A＜25 | slight |
| 3 | 25≤A＜50 | moderate |
| 4 | 50≤A＜80 | strong |
| 5 | 80≤A＜150 | very strong |
| 6 | 150≤A | severe |

Note: A indicate the annual average soil erosion in t hm^−2^ a^−1^
